# A case report of immune checkpoint inhibitor‐related myositis and cholangitis induced by pembrolizumab

**DOI:** 10.1002/ccr3.9153

**Published:** 2024-07-03

**Authors:** Takafumi Yamano, Masamitsu Hamakawa, Yoko Akaike, Tadashi Ishida

**Affiliations:** ^1^ Department of Respiratory Medicine Kurashiki Central Hospital Okayama Japan; ^2^ Department of Pathology Kurashiki Central Hospital Okayama Japan; ^3^ Present address: Department of Respiratory Medicine Osaka Red Cross Hospital Osaka Japan

**Keywords:** immune checkpoint inhibitor, myositis, pembrolizumab, sclerosing cholangitis

## Abstract

**Key Clinical Message:**

Rare but severe, immune‐related adverse events such as myositis and sclerosing cholangitis can occur with immune checkpoint inhibitors in lung cancer treatment. This case report highlights their co‐occurrence after pembrolizumab treatment, indicating the need for vigilance and management strategies in immune checkpoint inhibitors therapy.

**Abstract:**

Immune checkpoint inhibitors (ICI) are used in advanced treatment of lung cancer but can lead to immune‐related adverse events. ICI‐related myositis and cholangitis are rare, and their combination has not been previously reported. Here, we report the first case of ICI‐related myositis and sclerosing cholangitis. A patient with stage IV lung adenocarcinoma who received one cycle of pembrolizumab with cisplatin and pemetrexed developed myositis. Treatment with prednisolone improved the myositis, but the patient subsequently developed cholangitis. The patient did not respond to a regimen of prednisolone, mycophenolate mofetil, and azathioprine, and eventually died due to worsening lung cancer. An autopsy confirmed the presence of ICI‐related myositis and sclerosing cholangitis.

## Graphical abstract

Immune‐related adverse events such as myositis and sclerosing cholangitis are rare, but can occur with immune checkpoint inhibitors. The figure shows the co‐occurrence of these conditions.

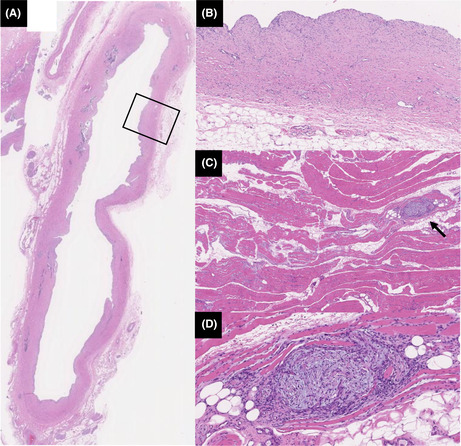

## 1 INTRODUCTION

Immune checkpoint inhibitors (ICIs) are effective against a variety of cancers, including lung cancer. They sustain T cell activation by inhibiting the activity of immune checkpoint molecules, including cytotoxic T‐lymphocyte‐associated protein 4, programmed cell death 1 (PD‐1), and programmed cell death ligand 1 (PD‐L1), resulting in increased activation of the immune system.^1^ Pembrolizumab is a humanized immunoglobulin G4 (IgG4) monoclonal antibody that targets PD‐1, and often results in various immune‐related adverse events, such as myositis and sclerosing cholangitis. Myositis and sclerosing cholangitis are rare immune‐related adverse events, occurring at a frequency of 0.3–1%^2^, ^3^, ^4^, ^5^ and 0.2%–4.5%.^6^, ^7^ Thus, no case of combined myositis and sclerosing cholangitis has been documented to date. Herein, we report the case of a patient with pembrolizumab‐induced ICI‐related myositis and sclerosing cholangitis.

### 1.1 Case history/examination

**FIGURE 1:**
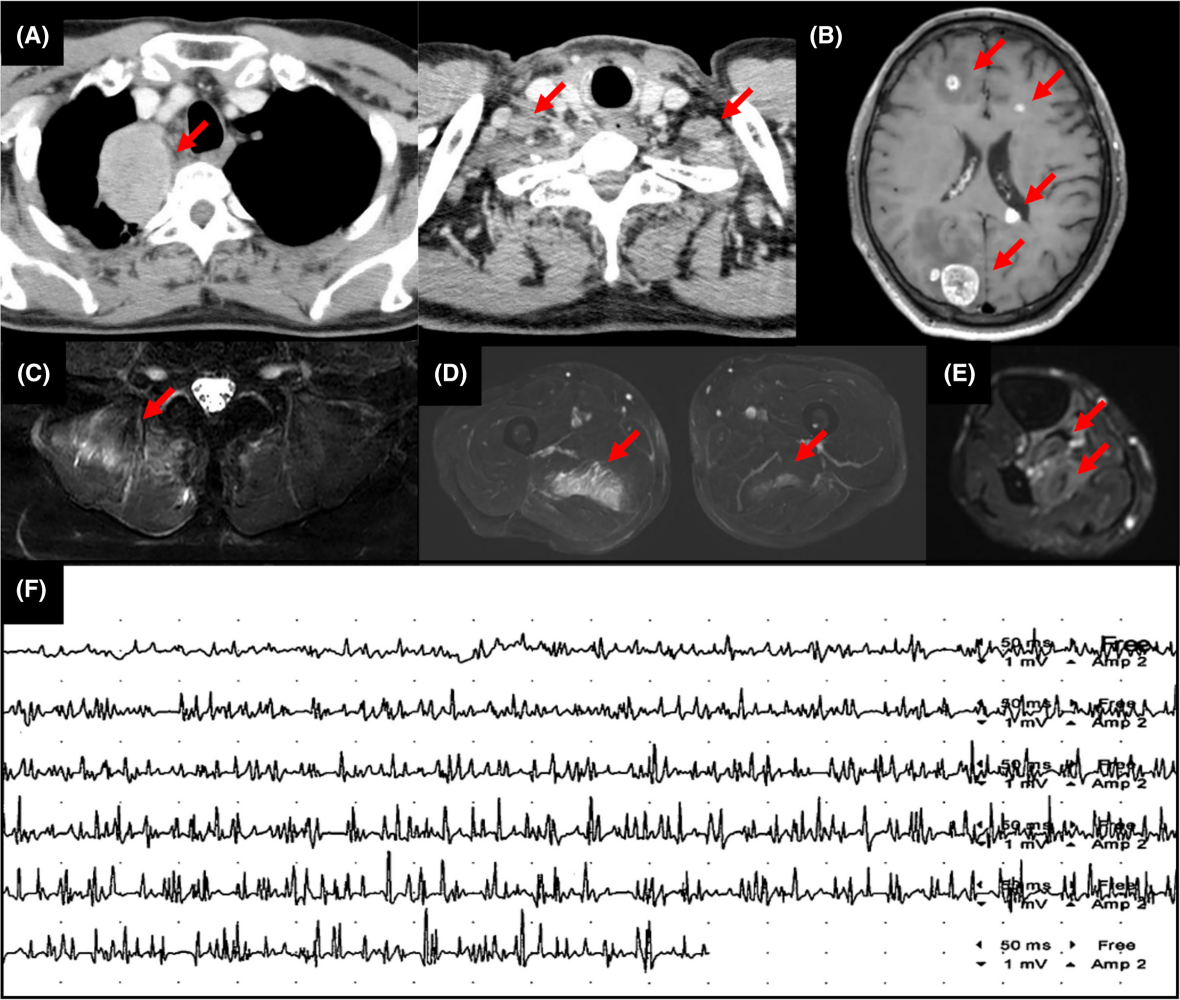
Diagnostic images of a 59‐year‐old male patient with lung adenocarcinoma. (A) Contrast‐enhanced computed tomography image shows lung cancer in the right upper lobe and bilateral supraclavicular lymphadenopathy. (B) Magnetic resonance imaging reveals multiple brain metastases. Axial STIR images shows hyperintense signals in the (C) right erector spinae muscles, (D) bilateral adductor magnus muscle, (E) right posterior tibial muscle, flexor digitorum longus muscle, and flexor hallucis longus (arrows). (F) Electromyography shows early recruitment and polyphasic, short‐duration, low‐amplitude motor unit action potentials.

A 59‐year‐old male patient with a history of smoking (Brinkman index, 780), and an Eastern Cooperative Oncology Group performance status score of 1, presented to our hospital with a 2‐month history of left visual field loss. The physical examination revealed left homonymous hemianopia but no other abnormal findings. Magnetic resonance imaging (MRI) of the head showed multiple brain metastases, and computed tomography (CT) revealed a 60 mm mass in the right upper lung, bilateral supraclavicular lymphadenopathy, which led to the diagnosis of lung adenocarcinoma cT3N3M1c stage IVB (Figure 1A,B). Pembrolizumab (200 mg/body intravenously every 3 weeks) with cisplatin (75 mg/m^2^ intravenously every 3 weeks) and pemetrexed (500 mg/m^2^ intravenously every 3 weeks) was administered, and the patient developed pain in his right thigh and lumbar region on day 10. Physical examination revealed tenderness in the right medial thigh, right lower leg, and right lumbar region, and the manual muscle testing score was 5, bilaterally in the upper and lower limbs and neck. Laboratory test results indicated an elevated inflammatory response and muscle enzyme levels, without troponin I or hepatobiliary enzymes elevation. However, autoimmune antibodies associated with myalgia gravis and myositis were not detected (Table 1) and his electrocardiogram findings were normal.

**TABLE 1:** Laboratory findings on day 10.

	Value	Reference range		Value	Reference range		Value	Reference range
Blood count			CK (IU/L)	3972	59–248	Anti‐Ro‐52 antibody	(−)	(−)
WBC (/μL)	7400	3300–8600	CK‐MB (ng/mL)	46.8	<5.0	Anti‐ARS antibody	(−)	(−)
Neutrophil (%)	75.2	38.0–77.0	Creatinine (mg/dL)	1.01	0.65–1.07	Anti‐MDA‐5 antibody	(−)	(−)
Lymphocyte (%)	12.4	15.0–53.0	Sodium (mEq/L)	135	138–145	Anti‐TIF‐1γantibody	(−)	(−)
RBC (×10^4^/μL)	411	4.35–5.55	Potassium (mEq/L)	4.2	3.6–4.8	Anti‐Mi‐2 antibody	(−)	(−)
Hb (g/dL)	11.9	13.7–16.8	TSH (μIU/mL)	2.13	0.38–5.38	Anti‐AChR antibody	(−)	(−)
Plt (×10^4^/μL)	19.2	16.0–36.0	FT4 (ng/dL)	0.96	0.70–1.48	Anti‐titin antibody	(−)	(−)
Biochemistry			Troponin I (ng/mL)	<0.01	<0.034	Anti‐Kv1.4 antibody	(−)	(−)
CRP (mg/dL)	33.3	0.00–0.14	CEA	5	<5.0			
TP (g/dL)	5.1	6.6–8.1	SLX	30.7				
Alb (g/dL)	2.1	4.1–5.1	CYFRA	16.4	<3.5			
T‐Bil (mg/dL)	0.3	0.4–1.5	SCC	0.6	<1.5			
AST (IU/L)	116	13–30	Immunology					
ALT (IU/L)	54	10–42	IgG (mg/dL)	646	861–1747			
ALP (IU/L)	180	10–42	IgG4 (mg/dL)	<5.0	11–121			
γ‐GTP (IU/L)	190	13–64	RF (IU/L)	5.5	<15			
LDH (IU/L)	337	124–222	ANA (titer)	<40	<40			

Abbreviations: AChR, acetylcholine receptor; Alb, albumin; ALP, alkaline phosphatase; ALT, alanine aminotransferase; ANA, anti‐nuclear antibody; ARS, aminoacyl‐tRNA synthetase; ASMA, anti‐smooth muscle antibody; AST, aspartate aminotransferase; CEA, carcinoembryonic antigen; CK, creatine kinase; CK‐MB, creatine kinase MB; CRP, C‐reactive protein; CYFRA, cytokeratin 19 fragment; FT4, free T4; Hb, hemoglobin; IgG, immunoglobulin; IgG4, immunoglobulin 4; LDH, lactate dehydrogenase; MDA‐5, melanoma differentiation‐associated gene 5; Plt, platelet count; RBC, red blood cell count; RF, rheumatoid factor; SCC, squamous cell carcinoma related antigen; SLX, sialyl Lewis‐x antigen; T‐Bil, total bilirubin; TIF1‐γ, transcription intermediary factor 1‐γ; TP, total protein; TSH, thyroid stimulating hormone; WBC, white blood cell count; γ‐GTP, gamma glutamyl transpeptidase.

MRI of the lower limbs and lumbar region showed high‐density T2 fat‐suppressed images of the bilateral adductor magnus, right tibialis posterior, and erector spinae muscles (Figure 1C–E). Electromyography (EMG) of the right adductor magnus muscle revealed findings indicative of myogenic changes, including early recruitment and polyphasic short‐duration low‐amplitude motor unit action potentials (Figure 1F). A biopsy of the right adductor magnus muscle was performed, but no abnormal findings were observed.

### 1.2 Differential diagnosis, investigations, and treatment

The MRI and EMG results were suggestive of myositis, whereas the normal muscle biopsy results was considered a sampling error. Therefore, the patient was diagnosed with ICI‐related myositis, and treatment with 60 mg (1 mg/kg) prednisolone (PSL) was initiated on day 18. The patient's creatinine kinase levels normalized and myalgia improved; thus, the PSL dose was gradually tapered. However, the patient experienced epigastric pain on day 58 when the PSL dose was reduced to 40 mg. Physical examination revealed epigastric and liver percussion tenderness; however, Murphy's sign was negative. Laboratory tests revealed elevated inflammatory response and hepatobiliary enzyme levels. The patient was using a proton pump inhibitor for gastric ulcer prophylaxis.

**FIGURE 2:**
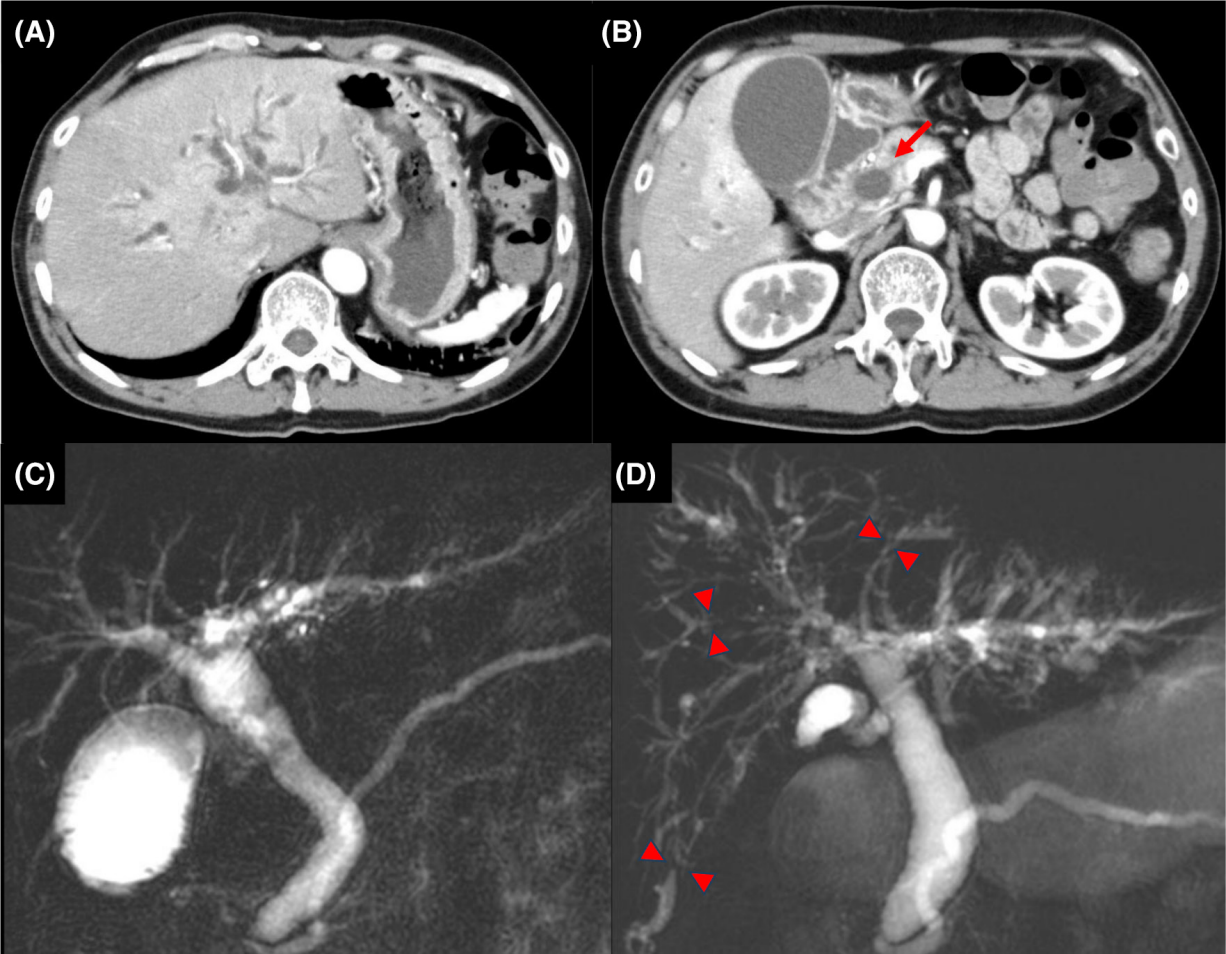
Abdominal contrast‐enhanced computed tomography (CECT) and magnetic resonance cholangiopancreatography (MRCP) findings. (A), (B) CECT image on day 58 after pembrolizumab shows intrahepatic bile duct dilatation, common bile duct dilation, and wall thickening (arrow). (C) MRCP on day 58 after pembrolizumab treatment shows common bile duct dilation. (D) MRCP on day 240 after pembrolizumab shows irregularly narrowed intrahepatic bile ducts (arrowheads) and dilation of the peripheral bile ducts.

Serum immunological markers, including anti‐nuclear antibody (ANA), antimitochondrial antibody (AMA), and IgG4, were within normal ranges (Table 2). Contrast‐enhanced CT revealed intrahepatic bile duct and common bile duct dilation and wall thickening. Magnetic resonance cholangiopancreatography (MRCP) was also performed, but there was no evidence of common bile duct stones or other factors that led to the obstruction (Figure 2A–C). Myositis showed a trend toward improvement, with the disappearance of the high signal in the T2 fat‐suppressed image on MRI of the right adductor magnus and right erector spinae muscles. Blood culture results were negative.

**TABLE 2:** Laboratory findings on day 58.

	Value	Reference range		Value	Reference range
Blood count			CK (IU/L)	15	59–248
WBC (/μL)	13,300	3300–8600	Creatinine (mg/dL)	0.75	0.65–1.07
Neutrophil (%)	82.6	38.0–77.0	Sodium (mEq/L)	140	138–145
Lymphocyte (%)	11	15.0–53.0	Potassium (mEq/L)	4.1	3.6–4.8
RBC (×10^4^/μL)	378	435–555	TSH (μIU/mL)	1.56	0.38–5.38
Hb (g/dL)	11.5	13.7–16.8	FT4 (ng/dL)	0.75	0.70–1.48
Plt (×10^4^/μL)	31.3	16.0–36.0	Amylase (IU/L)	36	44–132
Biochemistry			Lipase (IU/L)	23	13–55
CRP (mg/dL)	6.97	0.00–0.14	Immunology		
TP (g/dL)	5.2	6.6–8.1	IgG (mg/dL)	596	861–1747
Alb (g/dL)	2.3	4.1–5.1	IgG4 (mg/dL)	16	11–121
T‐Bil (mg/dL)	0.5	0.4–1.5	RF (IU/L)	4.0	<15
AST (IU/L)	61	13–30	ANA (titer)	<40	<40
ALT (IU/L)	1W04	10–42	AMA‐M2	(−)	(−)
ALP (IU/L)	262	10–42			
γ‐GTP (IU/L)	548	13–64			
LDH (IU/L)	243	124–222			

Abbreviations: Alb, albumin; ALP, alkaline phosphatase; ALT, alanine transaminase; AMA, anti‐mitochondrial antibody; ANA, antinuclear antibody; AST, aspartate transaminase; CK, creatine kinase; CRP, C‐reactive protein; FT4, free T4; Hb, hemoglobin; IgG, immunoglobulin; IgG4, immunoglobulin 4; LDH, lactate dehydrogenase; Plt, platelet count; RBC, red blood cell count; RF, rheumatoid factor; T‐Bil, total bilirubin; TP, total protein; TSH, thyroid stimulating hormone; WBC, white blood cell count; γGTP, gamma glutamyl transpeptidase.

Consequently, meropenem (1 g intravenously, three times daily) treatment was initiated for acute bacterial cholangitis, resulting in mild improvement of the hepatobiliary enzymes. However, the symptoms did not completely improve, and alanine aminotransferase (ALT) levels were elevated; therefore, ICI‐related cholangitis was suspected. The PSL dose was increased from 30 to 60 mg, mycophenolate mofetil (MMF) 1000 mg and ursodeoxycholic acid (UDCA) 300 mg were added on day 70, UDCA was increased to 600 mg/day on day 80, and MMF was increased to 2000 mg on day 87. C‐reactive protein and ALT levels showed a decreasing trend after the treatment initiation; however, relapse was observed when the PSL dose was reduced to 15 mg and MMF was switched to azathioprine (AZA) on day 147.

### 1.3 Outcome and follow‐up

**FIGURE 3:**
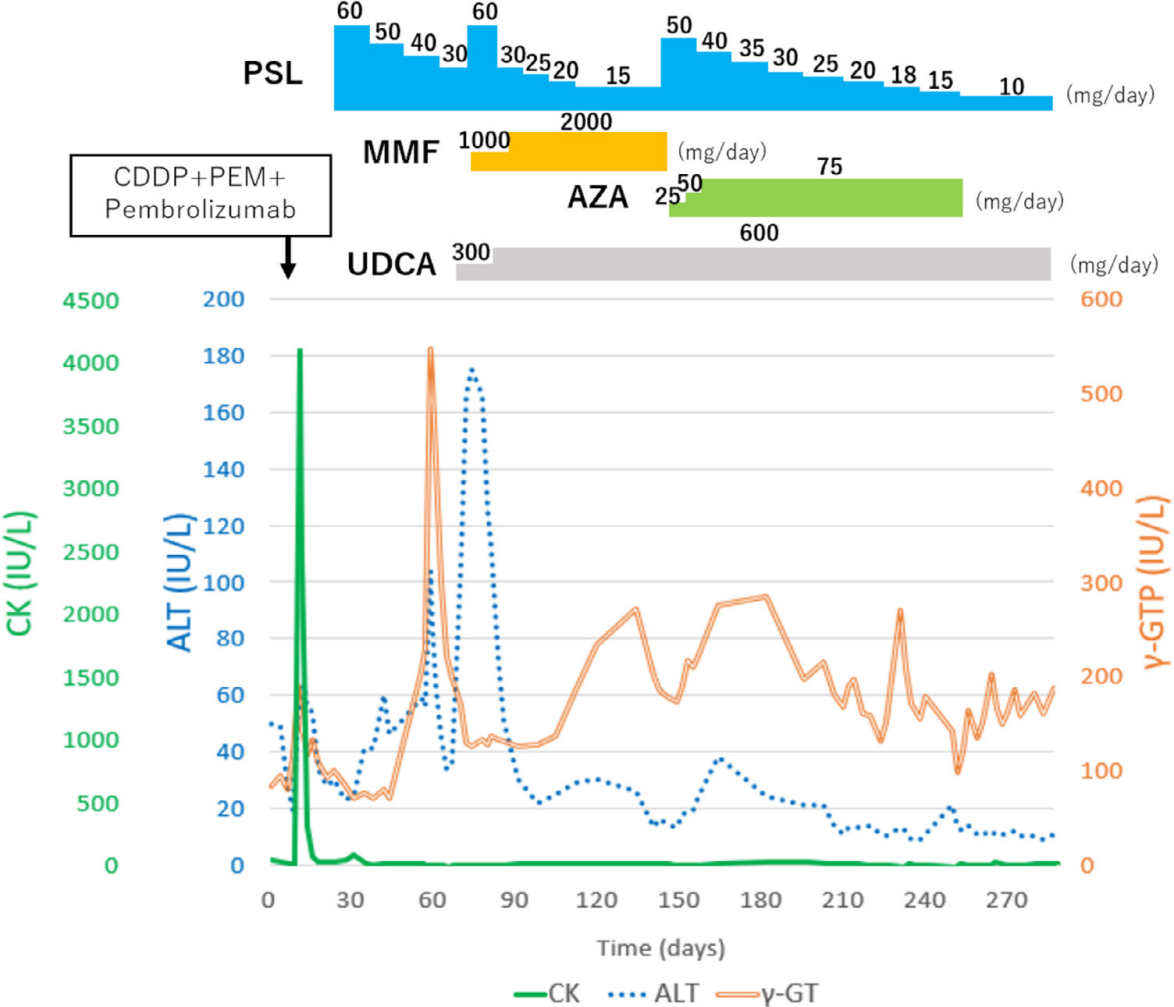
Clinical course of the present case of a 59‐year‐old male patient with pembrolizumab‐induced myositis and cholangitis. ALT, alanine aminotransferase; AZA, azathioprine; CDDP, cisplatin; CK, creatine kinase; MMF, mycophenolate mofetil; PEM, pemetrexed; PSL, prednisolone; UDCA, ursodeoxycholic acid; γ‐GTP, gamma glutamyl transpeptidase.

**FIGURE 4:**
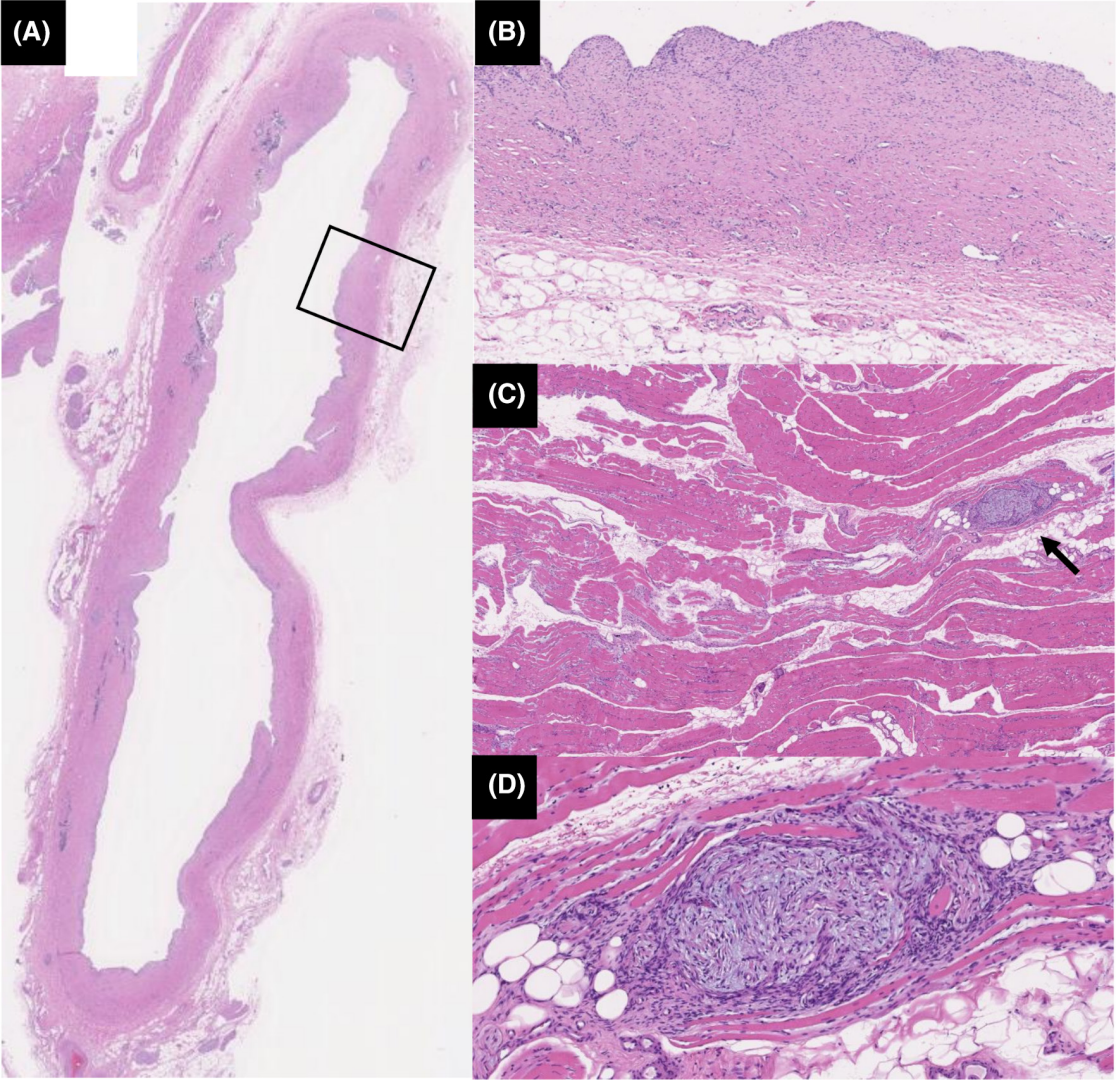
Pathological findings in the common bile duct and extensor digitorum longus muscles. (A) Hematoxylin and eosin (HE) staining of the common bile duct at low magnification (4×). (B) The image shows the area enclosed by the square at high magnification (40×). Dilatation of the common bile duct and thickening and fibrosis of the bile duct wall are seen. (C) HE staining of the extensor digitorum longus muscle at low magnification (10×) (arrow). (D) The image shows the area indicated by the arrow at high magnification (50×). Localized rhabdocyte shedding and fresh fibrosis with fibroblast proliferation are also noted.

Despite treatment modification, the hepatobiliary enzyme levels did not improve, and MRCP showed progressive narrowing of irregular intraductal bile ducts and peripheral bile duct dilatation (Figure 2D). Eventually, lung cancer progressed, and the patient died on day 289 (Figure 3). The autopsy revealed localized rhabdocyte shedding and fresh fibrosis with fibroblast proliferation in the right extensor digitorum longus muscle, dilatation of the common bile duct, and thickening and fibrosis of the bile duct wall (Figure 4). These findings were consistent with ICI‐related myositis and sclerosing cholangitis.

## 2 DISCUSSION

The present case involved a patient with lung adenocarcinoma who developed ICI‐related myositis and cholangitis. ICI‐related myositis is a rare condition, occurring at a frequency of 0.3%–1%.^2^, ^3^, ^4^, ^5^ Its onset ranges from 1 day to 80 weeks, with an average of 4–5 weeks after ICI administration.^3^, ^5^, ^8^, ^9^ Furthermore, ICI‐related myositis is more common in male patients and in individuals over 65 years old.^7^ The onset of myositis in the present patient was observed on day 10, which was slightly earlier than that reported previously.

Some patients with ICI‐related myositis are positive for anti‐TIF1γ, ANA, anti‐Ro 52, and anti‐PM/Scl antibodies but most are negative for autoantibodies.^10^ In contrast, over half of the patients with ICI‐related myopathy are positive for anti‐striational antibodies (anti‐titin and anti‐Kv1.4 antibodies).^11^ This patient tested negative for autoantibodies. Steroids are the first choice for ICI‐related myositis treatment, and MMF, AZA, cyclosporine, high‐dose intravenous immunoglobulin, and plasma exchange are used in patients with a poor response to treatment.^12^ The severity of the myositis in this patient was grade 2 in the Common Terminology Criteria for Adverse Events (ver 5.0), which recommends 0.5–1 mg/kg PSL^12^; therefore, treatment was initiated with 60 mg (1 mg/kg) PSL. The ICI‐related myositis is frequently complicated by myocarditis, which is refractory to treatment and has a poor prognosis.^3^, ^5^ However, the present patient had no complications of myocarditis and responded well to steroids.^10^, ^13^

The ICI‐related sclerosing cholangitis is a rare disease that occurs more frequently in male patients, with a frequency of 0.2%–4.5%,^6^, ^7^ and the average time to onset is 10–17.5 weeks.^6^, ^14^ In the present patient, disease onset occurred approximately 8 weeks after pembrolizumab treatment, similar to the timeframe in a previous report. The characteristics of ICI‐related sclerosing cholangitis include dilation of the extrahepatic bile ducts without obstruction, diffuse hypertrophy of the extrahepatic bile ducts and/or multiple strictures of the intrahepatic bile ducts, elevated hepatobiliary enzymes with a predominant biliary system and normal values for ANA, AMA, and IgG4.^6^, ^7^ The present patient had similar findings consistent with those previously reported. Biliary drainage was not performed in this patient owing to its poor efficacy^6^ and the associated risk of retrograde infection.^15^ Additionally, a liver biopsy was not performed because the patient was on PSL, and the findings could be masked. PSL (1–2 mg/kg) is the first choice of treatment for ICI‐related sclerosing cholangitis, although ICI‐related sclerosing cholangitis has been reported to have a moderate‐to‐poor response to steroids.^6^, ^7^ MMF, AZA, and tacrolimus are used to treat steroid‐resistant patients.^12^, ^16^, ^17^ MMF and AZA were used in this case owing to a poor response to steroids. However, the cholangitis could not be controlled and narrowing of the irregular intraductal bile ducts and peripheral bile duct dilatation were observed on MRCP. UDCA has been reported to be effective in some cases^6^, ^18^, ^19^, ^20^ and was used in this patient; however, it was ineffective.

Histopathology is characterized by the presence of CD8‐positive lymphocytic infiltrates and multifocal necrotic and regenerative fibers in ICI‐related myositis^21^, ^22^ and CD8‐positive T cell infiltrates in the bile ducts on liver biopsy in ICI‐related cholangitis.^7^ These findings were not observed in the present patient; however, fresh fibrosis consisting of a localized loss of rhabdomyocytes and fibroblast proliferation in the right flexor digitorum longus tendon and dilation, thickening, and fibrosis of the common bile duct were observed, which was consistent with ICI‐related myositis and sclerosing cholangitis after inflammation resolution. The reason for lack of intramuscular lymphocytic infiltration was considered to be a good response to treatment, as evidenced by the improvement in MRI findings. The lack of lymphocytic infiltration in the bile duct was considered unlikely to be the result of successful inflammatory treatment in view of disease progression on MRI, which may have been an end view of the inflammatory process.

The mechanism of ICI‐related myositis and sclerosing cholangitis remains unclear. However, it has been suggested that tumor‐reactive T cells, particularly CD8‐positive T cells, are stimulated by the immune checkpoint inhibitor and develop an autoaggressive response that results in immune related adverse events.^22^ The fact that CD8‐positive T cells are found in ICI‐related myositis^7^ and sclerosing cholangitis^21^, ^22^ suggests the activation of CD8‐positive T cells by immune checkpoint inhibitors causes myositis and sclerosing cholangitis.

## 3 CONCLUSION

This is the first report on the combined presence of ICI‐associated myositis and cholangitis. Further aggregation of cases is required to determine the risk factors for these combinations, response to treatment, and effective treatment options.

## AUTHOR CONTRIBUTIONS

**Takafumi Yamano:** Conceptualization; data curation; investigation; methodology; project administration; writing – original draft. **Masamitsu Hamakawa:** Writing – review and editing. **Yoko Akaike:** Writing – review and editing. **Tadashi Ishida:** Supervision.

## FUNDING INFORMATION

This research did not receive any specific grant from funding agencies in the public, commercial, or not‐for‐profit sectors.

## CONFLICT OF INTEREST STATEMENT

The authors have no conflicts of interest to declare.

## ETHICS STATEMENT

Not required for this case report.

## CONSENT

Written informed consent was obtained from the patient to publish this report in accordance with the journal's patient consent policy.

## Data Availability

The data that support the findings of this study are available from the corresponding author upon reasonable request.
